# A Randomized, Double-Blind, Sham-Controlled Trial of Transcranial Direct Current Stimulation in Attention-Deficit/Hyperactivity Disorder

**DOI:** 10.1371/journal.pone.0135371

**Published:** 2015-08-12

**Authors:** Camila Cosmo, Abrahão Fontes Baptista, Arão Nogueira de Araújo, Raphael Silva do Rosário, José Garcia Vivas Miranda, Pedro Montoya, Eduardo Pondé de Sena

**Affiliations:** 1 Postgraduate Program, Interactive Process of Organs and Systems, Federal University of Bahia, Salvador, Bahia, Brazil; 2 Spaulding Neuromodulation Center, Spaulding Rehabilitation Hospital, Harvard Medical School, Boston, Massachusetts, United States of America; 3 Bahia State Health Department (SESAB), Salvador, Bahia, Brazil; 4 Functional Electrostimulation Laboratory, Biomorphology Department, Federal University of Bahia, Salvador, Bahia, Brazil; 5 Postgraduate Program on Medicine and Human Health, School of Medicine, Federal University of Bahia, Salvador, Bahia, Brazil; 6 Institute of Physics, Federal University of Bahia, Salvador, Bahia, Brazil; 7 Research Institute on Health Sciences (IUNICS-IdisPa), University of the Balearic Islands, Palma, Spain; Los Angeles, UNITED STATES

## Abstract

**Background:**

Current standardized treatments for cognitive impairment in attention-deficit/hyperactivity disorder remain limited and their efficacy restricted. Transcranial direct current stimulation (tDCS) is a promising tool for enhancing cognitive performance in several neuropsychiatric disorders. Nevertheless, the effects of tDCS in reducing cognitive impairment in patients with attention-deficit/hyperactivity disorder (ADHD) have not yet been investigated.

**Methods:**

A parallel, randomized, double-blind, sham-controlled trial was conducted to examine the efficacy of tDCS on the modulation of inhibitory control in adults with ADHD. Thirty patients were randomly allocated to each group and performed a go/no-go task before and after a single session of either anodal stimulation (1 mA) over the left dorsolateral prefrontal cortex or sham stimulation.

**Results:**

A nonparametric two-sample Wilcoxon rank-sum (Mann-Whitney) test revealed no significant differences between the two groups of individuals with ADHD (tDCS vs. sham) in regard to behavioral performance in the go/no go tasks. Furthermore, the effect sizes of group differences after treatment for the primary outcome measures—correct responses, impulsivity and omission errors—were small. No adverse events resulting from stimulation were reported.

**Conclusion:**

According to these findings, there is no evidence in support of the use of anodal stimulation over the left dorsolateral prefrontal cortex as an approach for improving inhibitory control in ADHD patients. To the best of our knowledge, this is the first clinical study to assess the cognitive effects of tDCS in individuals with ADHD. Further research is needed to assess the clinical efficacy of tDCS in this population.

**Trial Registration:**

ClinicalTrials.gov NCT01968512

## Introduction

Attention-deficit/hyperactivity disorder (ADHD) is a neurodevelopmental disorder characterized by symptoms that may be related to an abnormal functioning of the prefrontal cortex involving inattention and impulsivity/hyperactivity [1–3]. According to previous studies, this condition is present in 5 to 9% of children, and it is estimated that up to 67% of these children will continue having symptoms in adulthood [2, 3]. One of the most common executive functions affected in adults with ADHD is inhibitory control [1, 4], i.e. the ability to inhibit an inconvenient behavior and/or to inhibit responses to distracting stimuli [4, 5]. Previous neuroimaging studies have also shown that brain activation is reduced in the prefrontal regions of these patients, suggesting compromised or reduced inhibitory control [6, 7]. Since response inhibition is essential for daily activities and behavioral adjustment, it is clear that enhancing inhibitory control may help improve these individuals’ quality of life [8]. Nevertheless, therapeutic approaches directed towards enhancing behavioral inhibition and improving other cognitive symptoms remain a challenge in ADHD [9].

Currently available standard pharmacological treatments are limited by factors such as their high cost and the presence of significant adverse events, which may restrict their clinical applicability [9, 10]. In this scenario, the development of new therapeutic approaches that would be safe, effective and accessible is crucial [11, 12]. Of the non-invasive brain stimulation techniques designed to modulate neural excitability, transcranial direct current stimulation (tDCS) represents an attractive option in view of its low cost, safety, feasibility and simple applicability [11]. This brain stimulation technique consists of the application of a weak electric current over the scalp through the use of conductive electrodes. Indeed, tDCS has already been shown to significantly modulate cortical excitability. The possibility of being able to modify prefrontal and striatal circuits [13, 14], the structure that is functionally compromised in ADHD patients [15, 16], may result in an improvement in executive functions [11, 15, 16]. Furthermore, it has been argued that anodal stimulation may increase cortical excitability by facilitating neuronal depolarization, whereas cathodal stimulation may decrease cortical excitability through hyperpolarization [17, 18].

Some clinical studies have broadened the potential usefulness of tDCS by evaluating the modulation of cognitive performance in healthy individuals and in several neuropsychiatric conditions [19–24]. Hsu et al. reported that anodal stimulation over prefrontal areas increased inhibitory control function in healthy subjects performing a stop-signal task [21]. Moreover, Boggio et al. reported an improvement in cognitive performance in a go/no-go task in patients with major depression following application of anodal stimulation over the left dorsolateral prefrontal cortex (DLPFC) [22]. Cognitive enhancement has also been evaluated in clinical trials in which a single session of anodal tDCS was applied over the left DLPFC, with a cathodal electrode positioned over the right DLPFC [23, 24]. It was found, for instance, that tDCS may enhance working memory significantly, as measured by performance in an N-back task [23]. Furthermore, a double-blind, controlled trial revealed an improvement in the attention of stroke patients as measured using a go/no-go task following a single session of anodal tDCS [24]. On the other hand, Loo et al. found that, compared to sham stimulation, anodal tDCS over the left DLPFC resulted in no significant differences in several cognitive functions evaluated in patients with depression [25]. However, in a subsequent study, the same author found that attention and working memory could be improved by applying tDCS with different parameters [26].

Although most of these previous tDCS trials already reported positive results insofar as the enhancement of cognitive performance is concerned, to the best of our knowledge this is the first study to assess the cognitive effects of tDCS in adults with ADHD. The objective of the present parallel, randomized, double-blind, sham-controlled trial was to investigate the efficacy of tDCS on the modulation of inhibitory control in adults with ADHD by applying a go/no-go task. The hypothesis to be tested was that anodal stimulation applied over the left DLPFC improves inhibitory control in adults with ADHD compared to the application of sham stimulation. In particular, tDCS was expected to increase the number of correct responses and reduce the number of impulsivity and omission errors compared to sham stimulation.

## Methods

This study was conducted at the Functional Electrostimulation Laboratory of the Federal University of Bahia (Salvador, Brazil) between May 2013 and April 2014. Methods were described following the CONSORT guidelines for randomized trial (S1 CONSORT Checklist).

### Participants

The target population consisted of individuals of 18 to 65 years of age who met the criteria for ADHD according to the Diagnostic and Statistical Manual of Mental Disorders, fourth edition, revised (DSM-IV-TR). An experienced psychiatrist confirmed diagnosis. Patients were screened using the Mini-International Neuropsychiatric Interview-Plus (MINI-Plus) and the Adult ADHD Self-Report Scale-18 (ASRS-18), a valid instrument based on DSM-IV criteria [27, 28]. This scale consists of two sections, each one containing nine questions that explore the frequency of the main symptoms of ADHD [27]. Participants with at least six symptoms classified as occurring often or very often in each section were recruited for this study. According to the eligibility criteria, individuals with major psychiatric disorders, alcohol abuse or who had used psychoactive substances in the previous 12 months were excluded from the study. Exclusion criteria also included individuals with cognitive impairment, defined by a score ≤ 24 in the Mini-Mental State Examination (MMSE) [29, 30], and those with any contraindication to tDCS such as metallic head implants or an implanted medical device.

Recruitment began in May 2013 through a targeted approach that consisted of contacting neuropsychiatric societies and associations, and sending invitation letters to neurologists and psychiatrists. In addition, patients were recruited using a broad-based strategy consisting of placing advertisements on the Internet and in social networks. Potential subjects were prescreened by email and telephone interviews. Eligible participants were screened once again during an on-site visit at the laboratory.

A computer-generated randomization method with a 1:1 permuted-block design was used to assign participants to the active or sham tDCS groups. An investigator who had no knowledge of any of the other aspects of this trial performed the randomization procedure and generated a list that was used to allocate patients, following their order of inclusion so as to ensure concealment (central randomization).

### Ethics Statement

The internal review board of the *Maternidade Climério de Oliveira*, Federal University of Bahia, approved this trial on 9th October 2012 under approval number 19311 (S1 and S2 Protocols). On the same date, the study was registered on the Brazil Platform, a national unified system for the registration of clinical trials and research involving human subjects. The final approval was issued on 8th November 2012 under CAAE number 07971612.2.0000.5543. All ongoing and related trials for this intervention are also registered.

Following the ethical principles for medical research involving human subjects laid out in the Declaration of Helsinki [31], all participants were provided with verbal and written details of the entire study protocol prior to undergoing any study procedure. All the individuals were capable of understanding and of providing their written informed consent, as this was one of the criteria for inclusion in this trial.

### Interventions

Participants underwent a single session of active or sham tDCS according to the group they were assigned to by block randomization. Anodal and cathodal electrodes were respectively placed at F3 and F4 electrode locations (10–20 EEG International System). These locations have proved ideal for the placement of stimulation electrodes in cognitive and behavioral studies [19–24]. In accordance with neuroanatomical parameters, F3 and F4 electrode locations correspond respectively to the left and right dorsolateral prefrontal cortex area. The intervention consisted of applying tDCS through two 5 x 7 cm saline-soaked sponges with conductive electrodes, delivered at 1.0 mA (current density 0.029mA/cm^2^) for 20 minutes using a stimulator (Quark Medical Products, model Nemesys 941, Brazil). Electrical current was ramped up for 30 seconds at the beginning of stimulation and decreased in the same fashion at the end of the session to avoid perception of fast transients [32].

In the case of the sham tDCS group, stimulation was initially applied for 30 seconds and the current was then switched off without the participant’s knowledge. This has been shown to be a reliable and effective sham-procedure to simulate the sensations observed at the beginning of active stimulation without modifying cortical excitability [11, 33, 34]. At the end of the session, participants were asked to guess which intervention they had received, active or sham, in order to assess the effectiveness of the blinding method. In addition to the participants, raters were also blinded with respect to which intervention was applied.

Safety was assessed through open-ended questions based on the tDCS adverse events questionnaire [35].

### Cognitive tasks

To test the effects of tDCS on cognition, subjects participated in a go/no-go task before and after the stimulation session. The task consisted of 150 trials to which subjects were asked to react by pressing the left button of a computer mouse with their right forefinger as soon as a previously designated target was presented on the screen (go stimulus). No behavioral reaction was required when no-go stimuli appeared. All the stimuli were displayed in black and white with the same dimensions on the computer screen for 650 milliseconds (msec), with 1000 msec as the interstimulus interval (ISI). Overall, 20% of the trials were defined as no-go trials. To avoid learning effects and to maintain a high level of attention, two versions of the same task were designed: one using images of letters and the other using pictures of different fruits as stimuli. All the participants performed both tasks before and after the stimulation session. The number of correct responses, impulsivity and omission errors were computed from each version of the go/no-go task.

### Procedure

After the screening procedure, participants were seated in a comfortable chair in a quiet room, approximately 50 cm from the computer screen. The order in which the two versions of the task (fruits vs. letters) were presented was counterbalanced among participants. Each subject participated in a single 2-hour visit with no subsequent follow-up.

### Statistical Analysis

The entire statistical analysis was conducted using the Stata statistical software program, version 13.0 (StataCorp LP, College Station, TX, USA). A sample size of 25 participants per arm was estimated for a power of 80% to identify a 50% difference in performance of the go/no-go task by participants in the active tDCS group and a 10% difference in the control group, with a bidirectional type I error probability of 0.05%. Considering the possibility of unexpected events such as dropouts, an attrition rate of 20% was estimated, resulting in a final sample of 60 patients. This sample was similar to those used in previous behavioral studies in which the tDCS technique was applied [26, 36].

The clinical and demographic characteristics of each group were assessed using descriptive statistical procedures such as calculating means and measures of dispersion (standard deviation [SD]). The characteristics of the participants in the two groups were compared at baseline using two-sample t-tests for continuous variables and the chi-square test for categorical variables.

The Shapiro-Wilk test was performed, histograms were constructed, and measures of central tendency and dispersion were calculated to test the assumption that distribution was normal. Raw dependent measures (the number of correct responses, impulsivity and omission errors) were compared using a nonparametric two-sample Wilcoxon rank-sum (Mann-Whitney) test for group differences (tDCS vs. sham) prior to stimulation. In addition, changes in the scores (the score after stimulation minus the score before stimulation) were computed and examined using this same procedure for the nonparametric data, and using two-sample t-tests for the results with normal distribution. To calculate the effect sizes of group differences, Cohen's d tests were performed using the mean and SD of the changes in the scores for correct responses and omission errors in each group (active vs. sham). For the changes in the scores for the impulsivity errors, the effect size of group differences was estimated using a nonparametric approach, dividing the Mann-Whitney U value squared by the group size [37]. Pearson’s chi-square was performed to assess the integrity of blinding. Statistical significance was set at 5% and all p-values were two-sided.

## Results

**Fig 1:**
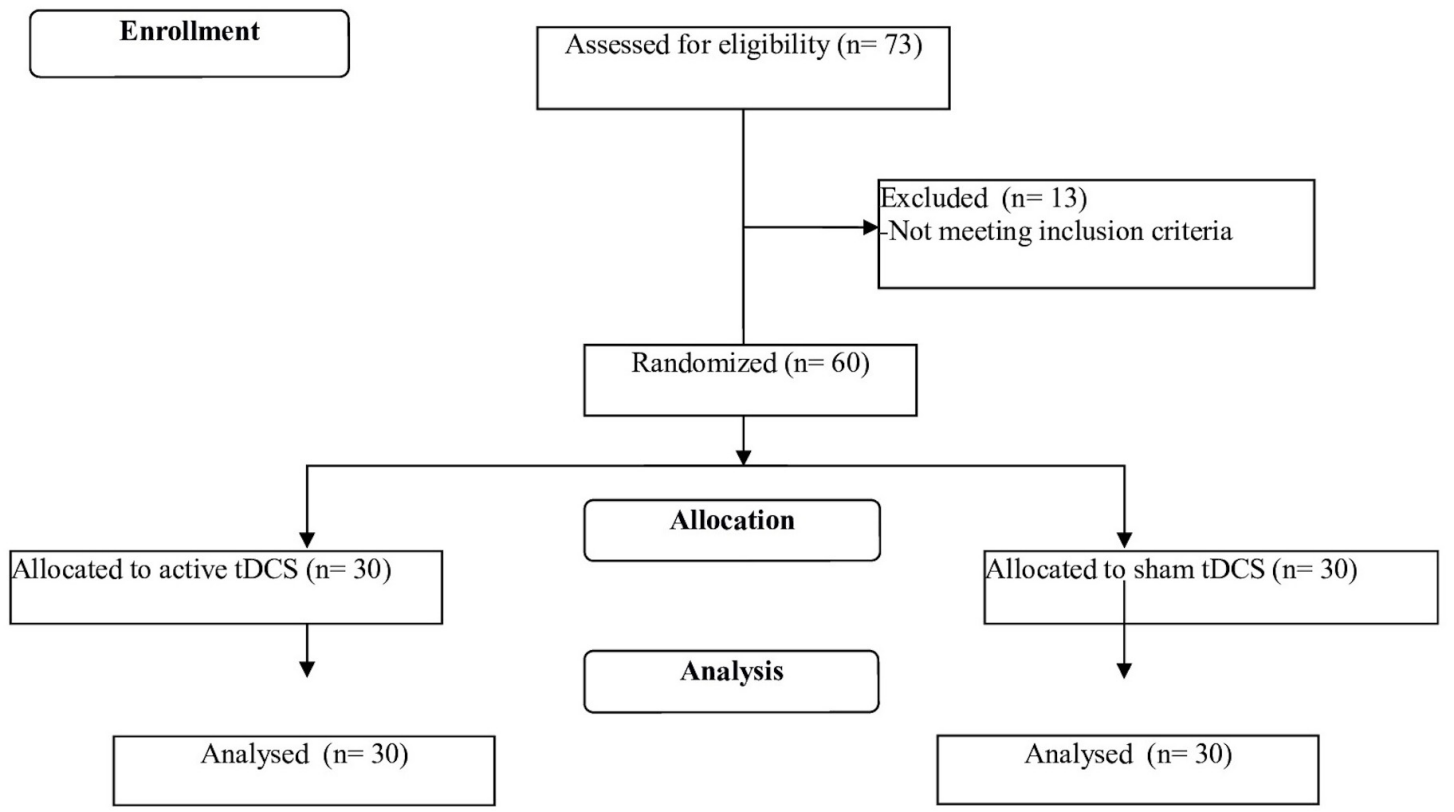
Study flowchart. Adapted from the CONSORT flow diagram (S1 Fig).

There were no statistically significant differences between the groups with respect to the demographic and clinical parameters (Table 1). In the present clinical trial, 73 participants were pre-screened, with 60 being enrolled to the study, 30 in each group (Fig 1). All the participants completed the entire protocol. Stimulation was well tolerated and no adverse events were reported.

**Table 1:** Demographic and clinical characteristics at baseline.

	Active Group	Sham Group
Age (years) ^a^	31.83 (11.55)	32.67 (10.37)
Male gender (%)	56.67	60.0
MMSE ^b^	28.77 (1.25)	28.93 (1.20)
Mean duration of disease (years)	21.77	22.90
Use of ADHD medication (%)	16.67	20.0
Types of ADHD (%) ^c^		
Combined inattentive/hyperactive/impulsive	76.67	70.00
Predominantly inattentive	20.00	23.33
Predominantly hyperactive/impulsive	3.33	6.67

^a^ Age presented as mean ± standard deviation (SD)

^b^ MMSE (Mini-Mental State Examination) described as mean ± SD

^c^ According to the criteria of the Diagnostic and Statistical Manual of Mental Disorders, fourth edition, revised (DSM-IV-R); ADHD, Attention-Deficit/Hyperactivity Disorder.

### Behavioral performance

Prior to the stimulation session, there were no differences between the groups (tDCS vs. sham) in either of the two tasks (go/no-go fruits and go/no-go letters). Indeed, there were no differences in correct responses (letters: U = -.28, p = .78; fruits: U = -.38; p = .71), impulsivity (letters: U = -1.53, p = .12; fruits: U = -.07; p = .94) or omission errors (letters: U = 1.32, p = .19; fruits: U = .65; p = .52).

Analyses of the effects resulting from the stimulation procedure also revealed no significant differences between the two groups (tDCS vs. sham) with regard to changes in the scores (the score after stimulation minus the score before stimulation). Therefore, there were no differences with respect to the correct responses (letters: t(58) = -.28, p = .78; fruits: t(58) = .40, p = .69), impulsivity (letters: U = .41, p = .68; fruits: U = .02, p = .98) or omission errors (letters: t(58) = -.27, p = .79; fruits: t(58) = -.14, p = .89) for either of the two tasks (Table 2).

**Table 2:** Performance on go/no-go tasks.

	Active Group	Sham Group	
	(Post—Pre)	(Post—Pre)	p-value
**Go/no-go (fruits)**			
Correct responses	8.40 (22.58)	10.57 (18.92)	.69 ^a^
Impulsivity errors	-5.54 (10.03)	-6.90 (10.40)	.98 ^b^
Omission errors	-2.87 (24.54)	-3.67 (21.16)	.89 ^a^
**Go/no-go (letters)**			
Correct responses	4.77 (18.83)	3.40 (18.48)	.78 ^a^
Impulsivity errors	-3.94 (13.34)	-1.27 (6.30)	.68 ^b^
Omission errors	-.83 (17.14)	-2.13 (20.41)	.79 ^a^

Values are described as means ± standard deviation

^a^ two-sample t-test comparing score changes (post-intervention minus pre-intervention scores) between groups, by tasks

^b^ Wilcoxon rank-sum (Mann-Whitney) U test comparing score changes (post-intervention minus pre-intervention scores) between groups, by tasks.

Furthermore, the effect size of group differences (active vs. sham) with regard to the changes in the scores was small for correct responses (letters: Cohen’s d = -.07; 95%CI,—.58 to .43; fruits: Cohen’s d = .10; 95%CI,—.40 to .61). It was also small for omission errors (letters: Cohen’s d = —.07; 95%CI, -.57 to .44; fruits: Cohen’s d = —.04; 95%CI, -.54 to .47) and for impulsivity errors (letters: $\hat{p}\;=\;0.00045$; fruits: $\hat{p}\;=\;0.000007$).

### Blinding integrity

When the blinding procedure used in the present study was tested, results showed that 43.33% of the participants who received the tDCS reported correctly that they were in the active group, whereas 70% of the participants who received the sham stimulation reported correctly that they were in the sham group. A chi-square analysis revealed that these differences were not statistically significant (χ^2^ = 1.15; p = .28).

## Discussion

To the best of our knowledge, this is the first clinical trial to assess the effects of tDCS on inhibitory control in ADHD patients. The present study examined behavioral performance in a go/no-go task before and after the application of tDCS for 20 minutes at 1 mA over the left dorsolateral prefrontal cortex (DLPFC) brain region (corresponding to the F3 electrode location). Although previous studies have shown that one single tDCS session at 1 mA is able to modulate cortical excitability [38, 39], the present results revealed that this intervention protocol exerted no significant effect on behavioral performance in the go/no-go task in ADHD patients. However, these findings conflict with the results of previous trials showing that one single session of tDCS at 2 mA over the left DLPFC is able to improve working memory performance [23]. Furthermore, Dockery et al. reported similar findings with one 15-minute session of tDCS at 1 mA [40]. One possible explanation for these differences between the present study and other previous ones could be associated with the moment at which tDCS was applied. For instance, preceding trials applied the tDCS protocol when subjects were simultaneously performing a cognitive task (online tDCS) [23, 39–41], whereas tDCS in the present study was applied when subjects were not performing any other task. In this context, Martin et al. compared the effects of anodal tDCS over the left DLPFC before and during a dual N-back working memory task [41]. They found an improvement in performance associated with stimulation during the cognitive task, suggesting an increase in the efficacy of tDCS when combined with cognitive performance. Thus, it is probable that the application of tDCS when subjects are actively involved in a cognitive task may activate more specific brain networks and may result in better performance than when they are at rest. The fact that no enhanced cognitive performance is observed after 10 sessions of tDCS when subjects are at rest [25] suggests that this factor could play a relevant role when assessing the clinical effectiveness of tDCS.

In addition, as previously shown in neuroimaging investigations [6, 7], it has to be taken into account that ADHD is a chronic neurodevelopmental disorder and that brain activation in the prefrontal regions is reduced in ADHD patients, even at rest. Therefore, although one single session of stimulation may enhance cortical excitability [23], this intervention may not have been strong enough to improve cognitive performance in this population. Furthermore, the selectivity of tDCS mechanisms remains unclear. Despite previous findings suggesting a target specific action [42, 43], the spreading of its modulatory effects have been reported [44–46] and this may explain the absence of any significant findings following a single session.

Previous studies have shown an improvement in response inhibition through the modulation of DLPFC activity using the tDCS technique [22, 36, 47]. In a factorial study, Fecteau et al. reported a decrease in risk taking, a behavior associated with increased inhibitory control, for bipolar montage with anodal stimulation over the left DLPFC delivered at 2 mA [47]. Similarly, Boggio et al. provided off-line anodal stimulation over the left DLPFC at an intensity of 2 mA and found an enhanced inhibitory response in a go/no-go task compared to sham [22]. The intensity and density of the current applied in these studies was 2 mA and 0.057mA/cm^2^ respectively, unlike those used in the present study in which tDCS was applied at 1 mA (current density 0.029mA/cm^2^), which may explain the different findings. However, the intensity used for this trial has already been shown to be effective in some neuropsychiatric disorders and its safety has already been well established. Since we are unaware of any previous studies in which this technique was applied to an ADHD population, we opted for a more conservative approach. In fact, no adverse effects were found in the present trial. In addition, it remains unclear whether higher intensity is associated with increased behavioral response [48–50].

The application of tDCS delivered at 1 mA has also been shown to ensure effective blinding [33, 51], whereas it has been suggested that studies using 2 mA of intensity are inappropriate insofar as the blinding procedure is concerned [52]. Subjects undergoing stimulation delivered at 2 mA report more sensory effects compared to tDCS at 1 mA. In the present study, stimulation was well tolerated and patients could not significantly differentiate between the active and sham intervention, indicating effective blinding. Naivety was another aspect that corroborated this, since none of the patients had ever received tDCS before.

Bipolar montage has already been successfully applied in previous trials conducted to assess cognitive performance [23, 24, 36]. An improvement was found in working memory following a single session of anodal stimulation over the left DLPFC, while a cathode electrode was placed over the contralateral DLPFC [23]. When electrodes were placed in a similar way to that used in this study (bifrontal with anode over the left DLPFC), an increase in inhibitory control was observed through a decrease in risk-taking behavior [36]. Although with this placement the cathodal electrode is also active and usually decreases cortical excitability [17, 18], possibly through the bifrontal montage, with the anode over the left DLPFC the improvement in inhibitory control could be explained by the balance obtained in the DLPFC areas. Despite these previous positive findings [23, 24, 36], a recent study reported that larger current densities are found in deeper regions for extracephalic montage when compared to cephalic placement [53]. Therefore, another explanation for the absence of statistically significant findings on cognitive performance in this population may involve the small current density in subcortical areas such as the striatum, a functionally compromised structure in ADHD [15, 16].

Another important aspect still under investigation is the spread of the current when using cephalic montage. A finite-element (FE) method has been used to investigate the current flow in the targeted areas in the brain [53, 54]. Using this mathematical model, different electrode montages were assessed by Neuling et al., including a bifrontal one [54]. Although with this placement the current was found to be focused in the frontal area, a small amount of current was found to have spread to the temporal and parietal areas, and a very weak current was present in the occipital cortex [54]. Considering that the montage examined by those investigators is similar to the bifrontal electrode placement applied in the present study, the spread of the current may also explain our findings.

## Conclusion

In conclusion, the findings of the present study provide no evidence of any improvement in inhibitory control after the application of tDCS over the left DLPFC compared to sham in adults with ADHD. To the best of our knowledge, this is the first clinical trial conducted to assess whether tDCS at rest could exert an effect on inhibitory control in adults with ADHD. Although previous studies have claimed that tDCS could be effective in modulating cortical excitability and in improving cognitive performance in healthy subjects and in specific neuropsychiatric disorders [19–24], further studies are necessary to clarify this issue. For future trials, better results may be achieved if cognitive training is provided together with tDCS. In addition, the number of intervention sessions should be increased and extracephalic montage used.

## Supporting Information

S1 CONSORT Checklist CONSORT checklist for randomized trial. (DOC)

S1 Fig CONSORT flow diagram. (TIF)

S1 Protocol Study protocol in English. (DOCX)

S2 Protocol Study protocol in Portuguese. (DOCX)
