# Supplementary figures and images for: A Randomized, Double-Blind, Sham-Controlled Trial of Transcranial Direct Current Stimulation in Attention-Deficit/Hyperactivity Disorder
Source: PLoS One. 2015 Aug 12;10(8):e0135371. doi: 10.1371/journal.pone.0135371 (PMC4534404; doi:10.1371/journal.pone.0135371)

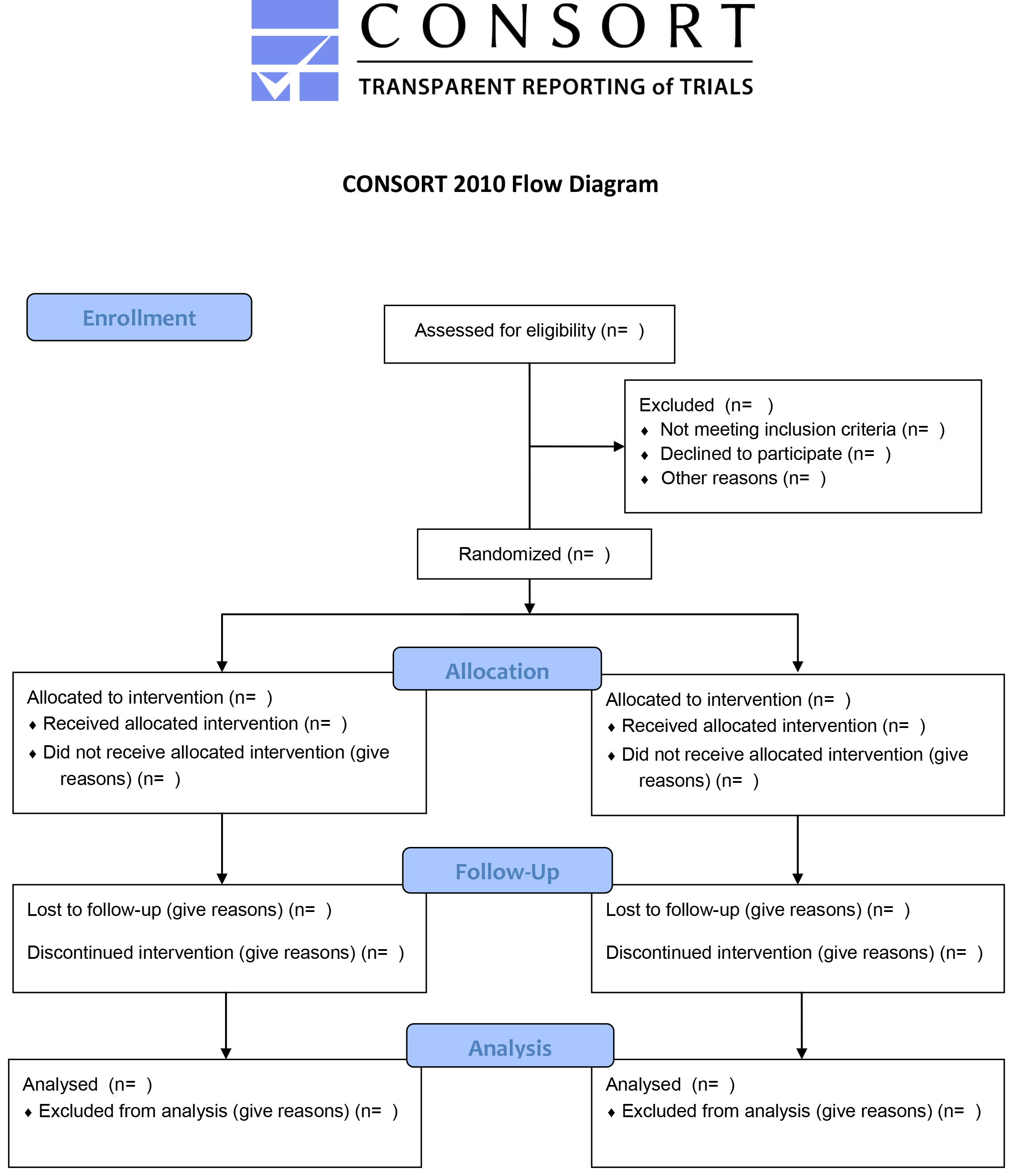

Supplement: S1 Fig — (TIF) [file pone.0135371.s002.tif]
