# Supplementary material for: A Randomized, Double-Blind, Sham-Controlled Trial of Transcranial Direct Current Stimulation in Attention-Deficit/Hyperactivity Disorder
Source: PLoS One. 2015 Aug 12;10(8):e0135371. doi: 10.1371/journal.pone.0135371 (PMC4534404; doi:10.1371/journal.pone.0135371)
Supplement: S1 Protocol — (DOCX) [file pone.0135371.s003.docx]

| **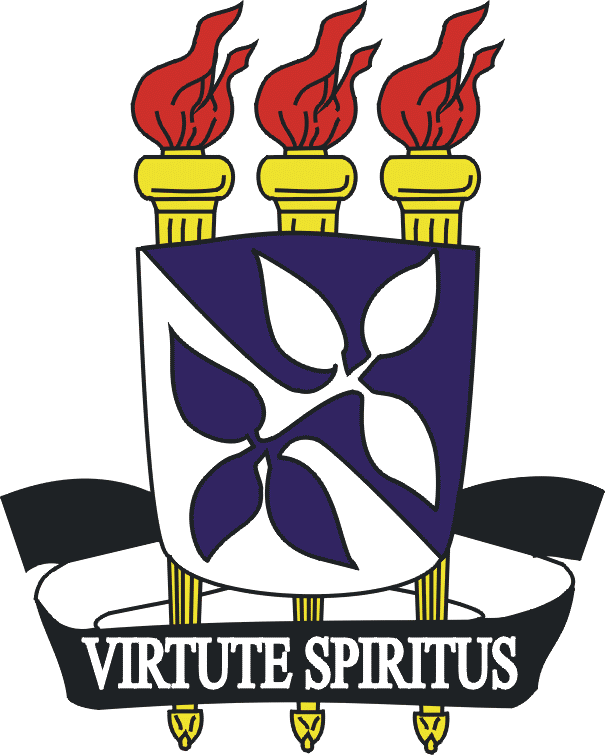** | **UNIVERSIDADE FEDERAL DA BAHIA**  **INSTITUTO DE CIÊNCIAS DA SAÚDE**  **PROGRAMA DE PÓS-GRADUAÇÃO EM PROCESSOS INTERATIVOS DOS ÓRGÃOS E SISTEMAS** | 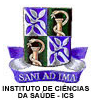 |
| --- | --- | --- |

**ASPECTOS NEUROBIOLÓGICOS DO TRANSTORNO DO DÉFICIT DE ATENÇÃO E HIPERATIVIDADE: CONTRIBUIÇÃO DA ESTIMULAÇÃO TRANSCRANIANA POR CORRENTE CONTÍNUA NO CONTROLE INIBITÓRIO**

**CAMILA SOUZA ALVES COSMO**

Projeto apresentado ao Comitê de Ética em Pesquisa da Maternidade Climério de Oliveira, Universidade Federal da Bahia, para apreciação e parecer, desenvolvido sob a orientação do Prof. Dr. Eduardo Pondé de Sena.

**Salvador, 2012**

**ÍNDICE**

| ÍNDICE DE ABREVIATURAS E SIGLAS | 03 |
| --- | --- |
| I. OBJETIVOS | 04 |
| I. a. GERAL | 04 |
| I. b. ESPECÍFICOS | 04 |
| II. FUNDAMENTAÇÃO TEÓRICA | 04 |
| III. MATERIAIS E MÉTODOS | 07 |
| IV. Aspectos éticos | 11 |
| V. VIABILIDADE TÉCNICA, CIENTÍFICA E FINANCEIRA | 11 |
| VI. RELEVÂNCIA | 12 |
| VII. CRONOGRAMA | 12 |
| VIII. REFERÊNCIAS | 12 |

**Índice de ABREVIATURAS E siglas**

AAQoL - Questionário de Qualidade de Vida em Adultos com TDAH;

ABENEPI - Associação Brasileira de Neurologia, Psiquiatria e Profissões Afins;

APB - Associação Psiquiátrica da Bahia;

ASRS - *Adult Self-Report Scale;*

CPC-SSP - Centro de Pesquisas Clínicas do Sanatório São Paulo;

DSM-IV-TR - Manual Diagnóstico e Estatístico de Transtornos Mentais- 4ºedição, revisão textual;

EEG – eletroencefalografia;

EEGQ – eletroencefalografia quantitativa;

ETCC - Estimulação Transcraniana por Corrente Contínua;

mA - Miliampère;

MEEM - Mini-exame do estado mental;

MINI PLUS - *Mini International Neuropsychiatric Interview Brasilian;*

SNB *-* Sociedade de Neurologia da Bahia;

TCLE - Termo de Consentimento Livre e Esclarecido;

TDAH - Transtorno de Déficit de Atenção e Hiperatividade.

**I. OBJETIVOS**

##### I.a. Geral

Avaliar os aspectos neurobiológicos associados ao Transtorno do Déficit de Atenção e Hiperatividade (TDAH) e a contribuição da estimulação transcraniana por corrente contínua (ETCC) na adequação do controle inibitório.

**I.b. Específicos**

**I.b.1.** Avaliar a contribuição da estimulação transcraniana por corrente contínua, comparando-a com intervenção *sham*, na modulação do controle inibitório em adultos com TDAH através de tarefas Go / No go;

**I.b.2.** Examinar os aspectos neurobiológicos evidenciados através da atividade elétrica cerebral observada por análise de poder de bandas de frequência em pacientes com TDAH, usando eletroencefalografia quantitativa;

**I.b.3.** Realizar a Reconstrução da Rede Funcional Cerebral, de cada participante, baseando-se nos dados do EEG quantitativo, através de modelagem matemático-computacional;

**I.b.4**. Registrar os indicadores de qualidade de vida em adultos com Transtorno de déficit de atenção e hiperatividade, através do Questionário de Qualidade de Vida em Adultos com TDAH (AAQoL);

**II.** **FUNDAMENTAÇÃO TEÓRICA**

O desenvolvimento humano compreende fases e esferas de exposição ambiental e social que influenciadas por aspectos fisiológicos e culturais constroem o escopo psíquico e físico de um indivíduo. Nesta ambiência, o transtorno de déficit de atenção e hiperatividade (TDAH), enquanto uma condição expressa por hiperatividade, impulsividade e redução da atenção sustentada e dirigida, pode colaborar para o comprometimento do desenvolvimento sócio-afetivo do indivíduo, se não houver uma intervenção precoce e efetiva [1,2].

O TDAH apresenta taxas de prevalência, em estudos americanos, de 5 a 9% na infância, principalmente em escolares, e entre estes 67% mantém a sintomatologia na vida adulta, repercutindo no desenvolvimento psicossocial, profissional e emocional [3- 6]. Em um recente estudo nacional, Arruda e colaboradores observaram uma prevalência de TDAH entre crianças e adolescentes de 4,4%, em acordo com dados da literatura mundial [7]. Entretanto, apesar do significativo impacto social, parte destes pacientes permanece sem diagnóstico, e portanto sem a intervenção terapêutica devida. O diagnóstico baseia-se na avaliação clínica e identificação de critérios estabelecidos pelo Manual Diagnóstico e Estatístico de Transtornos Mentais- 4º edição-revisão textual (DSM-IV-TR), como critérios de desatenção, de hiperatividade, de impulsividade e critérios gerais [1- 4, 6].

O quadro clínico origina-se, possivelmente, de desordens no córtex pré-frontal e de suas conexões subcorticais [1, 6, 8]. Esta suposição é respaldada pela hipótese de que se trata de um distúrbio neurobiológico, evidenciado por déficit funcional em neurotransmissores- como a dopamina e noradrenalina, e déficit funcional no lobo frontal, desencadeando os sintomas observados nos portadores de TDAH, tais como impulsividade, dificuldade em inibir comportamentos distratores, em planejar e executar tarefas, em direcionar a atenção e manter a concentração [6]. Estas observações evidenciam o comprometimento de importantes funções executivas em indivíduos com TDAH [2-4, 6, 8, 9].

O córtex pré-frontal é a principal área encefálica responsável pelas funções executivas [10]. Estas correspondem a habilidades neurocognitivas de planejamento e execução de ações intencionais e auto-organizadas [8, 9, 11]. Entre as funções executivas, o controle inibitório é um dos aspectos mais comprometidos no transtorno de déficit de atenção e hiperatividade [6]. Este corresponde à condição de inibir um comportamento inconveniente ou inadequado ou/e inibir reposta à estímulos distraidores [6, 8, 12]. Um transtorno do controle inibitório, portanto, prejudica significativamente o desempenho social, afetivo e cognitivo do indivíduo, seja por impossibilitar a interrupção de uma ação inapropriada ou por não intervir na interrupção de um estímulo que descontinue respostas adequadas [2, 3, 6].

Há evidências de uma associação fisiológica entre o córtex pré-frontal e o controle inibitório [8, 12, 13]. Alterações nesta estrutura anatômica possivelmente são responsáveis por um prejuízo no controle inibitório, evidenciado comportamentalmente por ações que refletem inconsequência, imprevisibilidade, intolerância à espera, respostas rápidas e inconsistentes, eventuais exposições a situações de risco, e aceitação de múltiplas responsabilidades e tarefas, simultaneamente, com consequentes abandonos posteriores [8, 13].

Esta relação anatômica e compreensão dos mecanismos neurobiológicos compõem um arcabouço fisiológico que sustenta a hipótese de que a ativação da área pré-frontal resultará em um maior e mais adequado controle inibitório, minimizando as repercussões sócio-afetivas associadas a esta condição.

A estimulação transcraniana por corrente contínua (ETCC) é uma técnica neuromodulatória simples e bem conhecida [14, 15], que utiliza correntes eléctricas de baixa tensão, para aumentar ou diminuir a excitabilidade neuronal da área estimulada [14, 16]. Esta é uma intervenção não-invasiva e segura, possuindo efeitos adversos pouco frequentes e inexpressivos, caracterizados por leve desconforto local, prurido, parestesia e/ou cefaleia, de curta duração [16]. A aplicação desta técnica e a avaliação dos seus efeitos será realizada por observação clínica considerando como parâmetros uma redução na sintomatologia avaliada através de tarefas neuropsicológicas- Go/ No go, e por avaliações neurofisiológicas como eletroencefalograma quantitativo (QEEG) e reconstrução de redes cerebrais. Neste ensaio clínico, estas técnicas irão avaliar provável ativação do córtex pré-frontal após ETCC, e investigarão a hipótese de aumento no controle inibitório e suas consequências comportamentais. Será aplicada estimulação anódica de 1mA, objetivando aumento da excitabilidade do córtex dorsolateral pré-frontal, de acordo com a correlação anatómica e fisiológica acima mencionadas. Ao final, espera-se um melhor ajustamento das respostas comportamentais associados ao controle inibitório que compromete, em última instância, a qualidade de vida de indivíduos com TDAH [16].

O conceito de qualidade de vida é complexo, amplo e pode ser entendido em diferentes contextos: biológicos, econômicos, culturais e sociais [19]. Considerando a abordagem deste estudo- a perspectiva biológica, qualidade de vida expressa a condição dos indivíduos compreenderem a doença, seus desafios e inadequações comportamentais de uma forma clara que lhes permita definir estratégias de adaptação.

Considerando os métodos terapêuticos disponíveis, fármacos como o metilfenidato e lisdexanfetamina contribuem para melhor controle sintomático [20]. No entanto, esses medicamentos apresentam efeitos colaterais importantes, com ocorrência imprevisível, tais como insônia, perda de apetite, dor de cabeça, espasmos musculares, irritabilidade, ansiedade, eventos cardiovasculares, entre outros, que podem eventualmente resultar em interrupção do tratamento [20-24]. Neste contexto, novas estratégias terapêuticas com perfil simples e seguro, como a estimulação transcraniana por corrente contínua, são essenciais.

Objetivamos neste estudo, investigar a eficácia da ETCC sobre o córtex pré-frontal na modulação do controle inibitório em adultos com TDAH, evidenciada através de parâmetros comportamental e neurofisiológicos, mencionados anteriormente. Esperamos observar um aumento na resposta inibitória em pacientes com TDAH, podendo resultar em uma maior capacidade para inibir comportamentos inadequados, através da aplicação de uma técnica de baixo custo, segura e não-invasiva.

**III. MATERIAIS E MÉTODOS**

**III.a**. **Desenho do Estudo**

Trata-se de um estudo paralelo e transversal que incluirá etapas descritivas e analíticas destinadas a investigar os aspectos neurobiológicos de pacientes com transtorno de déficit de atenção e hiperatividade, e a resposta destes parâmetros, associados ao controle inibitório, à modulação por estimulação transcraniana por corrente contínua.

**III.b. População**

Pacientes com transtorno de déficit de atenção e hiperatividade serão recrutados através de carta e e-mail para a Associação Psiquiátrica da Bahia (APB), Sociedade de Neurologia da Bahia (SNB) e Associação Brasileira de Neurologia, Psiquiatria e Profissões Afins (ABENEPI)-capítulo Bahia. Também serão contactados, através de correspondência e telefone, profissionais especialistas em TDAH, convidando-os a encaminhar prováveis pacientes ao estudo. Serão ainda publicados anúncios em internet e redes sociais, no período de outubro de 2012 a dezembro de 2012, de acordo com os critérios de inclusão.

**III.b.1. Critérios de inclusão**

-Habilidade para entender e assinar o formulário de consentimento informado;

-Diagnóstico de transtorno de déficit de atenção e hiperatividade, fundamentado no DSM-IV-TR e *Adult Self-Report Scale* (ASRS);

-Residentes na Bahia;

-Maiores de 18 anos.

**III.b.2. Critérios de exclusão**

-Comorbidades psiquiátricas tais como: esquizofrenia e transtorno bipolar;

-Incapacidade de compreensão dos questionários utilizados (comprometimento cognitivo- escore ≤ 24 no Mini Exame do Estado Mental) ou não alfabetizados;

-Abuso de substâncias psicoativas ou álcool, exceto nicotina e cafeína, nos últimos 12 meses.

**III.b.3. Tamanho da amostra**

Trata-se de amostra randomizada de indivíduos com TDAH recrutados através de Internet, redes sociais, além de contato com especialistas e sociedades especializadas em neurologia, psiquiatria e áreas afins, no período do estudo. Este tamanho amostral foi calculado estimando-se uma diferença de proporção entre o grupo de ativo e controle de 40%, na modificação do desempenho da tarefa Go/No go, antes e após as intervenções.

Este cálculo foi realizado utilizando-se o programa estatístico Stata 12.0, considerando um erro alfa de 0,05 e um poder de 0,80, obtendo-se uma amostra de 25 sujeitos em cada grupo, intervenção e controle. Considerando possíveis fatores inesperados, assumimos uma taxa de desistência de 20%, alcançando uma amostra final de 60 indivíduos.

**III.c. Variáveis do Estudo**

**III.c.1. Variáveis dependentes**

As seguintes variáveis serão avaliadas: desempenho nas tarefas neuropsicológicas (Go/ No go); análise de poder de faixas de bandas de frequências através de EEGQ; e modelo de reconstrução de redes cerebrais, de cada indivíduo, também através de EEGQ.

**III.c.2. Variáveis independentes**

Estimulação transcraniana por corrente contínua e procedimento *sham*.

**III.d. Descrição dos Procedimentos**

Após a triagem através de entrevista psiquiátrica, explicação sobre todas as etapas do estudo, e concordância expressa através de assinatura do termo de consentimento, os pacientes com TDAH serão submetidos a uma avaliação cognitiva através do mini-exame do estado mental (MEEM), ao Mini International Neuropsychiatric Interview Brazilian (MINI PLUS), Adult Self-Report Scale (ASRS), questionário sobre a qualidade de vida em adultos com TDAH (AAQoL), e uma entrevista através de um questionário. Este roteiro de avaliação é um instrumento com base nos critérios de diagnóstico de TDAH baseados no DSM-IV-TR, e consiste em questões epidemiológicas e clínicas.

Os indivíduos serão alocados em dois grupos através de randomização em bloco, objetivando uma distribuição equitativa entre os grupos (30 indivíduos em cada). Serão compostos blocos de 10 participantes, totalizando seis blocos. Para compor cada um dos grupos serão convocados 5 sujeitos de cada bloco.

Após esta etapa inicial, a atividade elétrica cerebral será registrada, durante 5 minutos, através de EEG com 32 canais posicionados no couro cabeludo em acordo com o sistema internacional 10-20. A gravação será com os indivíduos em repouso, no primeiro minuto com os olhos abertos, fixos em um ponto específico, e nos últimos 4 minutos com os olhos fechados. Em seguida, os participantes serão convidados a realizar duas tarefas do tipo Go/ No go, no computador, adaptadas da versão original, a primeira utilizando como alvo uma fruta, e a segunda uma letra. Após as tarefas cognitivas, os indivíduos serão submetidos a estimulação ativa ou *sham*, de acordo com a randomização prévia.

Os 30 participantes do grupo de intervenção serão submetidos à ETCC com voltagem de 1mA, ânodo em córtex pré-frontal dorsolateral esquerdo e cátodo na região equivalente à direita. Nos 30 indivíduos do grupo controle (sham) serão colocados eletrodos em posições idênticas e simulada a estimulação, porém o aparelho será ligado por apenas 30 segundos, para que o paciente tenha a sensação inicial, sendo desligado em seguida. A intervenção e o procedimento placebo ocorrerão em 1 única sessão para avaliação do efeito imediato, com duração de 20 minutos.

No final da intervenção, os indivíduos irão realizar novamente duas tarefas Go/ No go (selecionadas aleatoriamente para evitar o efeito de aprendizagem), e irão submeter-se a outro registro de EEG, com as mesmas características técnicas anteriores.

Este estudo será realizado no Laboratório de Eletroestimulação Funcional da Universidade Federal da Bahia.

**III.e. Plano de análise estatística**

1ª etapa: descrições sócio-demográfica, clínica e epidemiológica dos grupos, utilizando os procedimentos habituais da estatística descritiva, como cálculo de frequências, medidas de tendência central e de dispersão;

2ª etapa: as referidas características serão comparadas entre os grupos, antes da intervenção, utilizando análise de variância (ANOVA) para variáveis contínuas e teste do qui-quadrado para variáveis categóricas;

3ª etapa: teste de Shapiro-Wilk será realizado para avaliar a normalidade das variáveis dependentes;

4ª etapa: análise de amostras pareadas e independentes, utilizando o teste t para comparação dentro de cada grupo e entre os grupos de intervenção, ou aplicação de testes não-paramétricos equivalentes (de acordo com os resultados do teste de Shapiro-Wilk), antes e depois da intervenção;

5ª etapa: teste do qui-quadrado de Person para avaliar a eficácia da técnica de cegamento, comparando entre os grupos.

Estas análises serão realizadas através do programa estatístico *Stata*, versão 12.0 para *Windows*. Os resultados serão considerados estatisticamente significantes se valor de p ≤ 0,05.

**III.f. Limitações do estudo e estratégias para evitar viés**

**IV.f.1. Erro de seleção:** serão utilizados critérios objetivos e já amplamente empregados para ao diagnóstico de transtorno de déficit de atenção e hiperatividade, sendo a possibilidade de inclusão de indivíduos com outro diagnóstico improvável.

**IV.f.2.** **Erro de aferição:** as escalas utilizadas são instrumentos com validade e precisão reconhecidos e aceitáveis.

**IV. ASPECTOS ÉTICOS**

O estudo segue estritamente os preceitos de ética em pesquisa envolvendo seres humanos, em acordo com a declaração de Helsinki. Todos os participantes serão informados sobre a natureza do estudo e todos os procedimentos. Serão incluídos apenas os que formalizarem consentimento de participação por escrito, em conformidade com a resolução 196/96 do conselho nacional de saúde (Brasil), através do termo de consentimento livre e esclarecido- TCLE [25]. Será necessária a assinatura de uma testemunha considerando aqueles pacientes incapazes de assinar o termo de consentimento livre e esclarecido.

**V. VIABILIDADE TÉCNICA, CIENTÍFICA E FINANCEIRA**

O objetivo deste estudo é avaliar aspectos neurobiológicos associados ao transtorno de déficit de atenção e hiperatividade, e a contribuição da estimulação transcraniana por corrente contínua na modulação do controle inibitório e de parâmetros neurofisiológicos, demonstrados através de avaliações cognitivas (Go/ No go), EEGQ e do modelo de reconstrução de redes cerebrais. Este estudo não irá interferir com o tratamento habitual dos indivíduos com TDAH, para evitar quaisquer prejuízos associados à interrupção do tratamento. Através da investigação da eficácia de uma técnica simples, aplicada sobre o córtex pré-frontal, espera-se aumentar o controle inibitório em indivíduos com TDAH. Também esperamos que o presente ensaio clínico contribua cientificamente para o desenvolvimento de métodos de avaliação neurofisiológicos deste transtorno, e para a avaliação da aplicabilidade de uma técnica de baixo custo, não-invasiva e segura para a otimização da resposta inibitória nesta população.

O orçamento do projeto foi revisado e aprovado pelo Prof. Dr. Eduardo Pondé, e inclui os custos dos materiais utilizados na coleta de dados. O laboratório onde a coleta de dados será realizada, já dispõe dos equipamentos necessários, tais como aparelhos de ETCC, EEG com 32 canais, computadores, além de eletrodos, cabos e esponjas, sendo estimada apenas uma despesa muito baixa com materiais como questionários, o que explica o baixo orçamento e reitera a viabilidade financeira deste projeto.

**VI. RELEVÂNCIA**

Os resultados deste ensaio clínico nos permitirão avaliar os aspectos comportamentais e neurobiológicas do transtorno de déficit de atenção e hiperatividade, além de observar os parâmetros sócio-demográficas e clínico-epidemiológicos desta população.

Também será avaliada, como mencionado previamente, uma técnica de baixo custo, simples e não-invasiva para a modulação do controle inibitório.

Segundo nosso conhecimento, este estudo será o primeiro a avaliar os efeitos cognitivos e neurofisiológicos da ETCC em pacientes com TDAH. A longo prazo, esperamos que os nossos resultados possam reforçar programas abrangentes de intervenção e abordagem multidisciplinar em pacientes com TDAH.

**VII. CRONOGRAMA**

Após a aprovação do projeto, por este Comité de Ética em Pesqusia, o recrutamento será iniciado em maio de 2013, com coleta de dados a partir de junho de 2013. Espera-se que a análise inicial dos dados ocorra aproximadamente em março de 2014, seguida de elaboração da tese e respectivos artigos científicos, durante o segundo semestre de 2014.

**VIII.** **REFERÊNCIAS**

1. Lange, K. W. et al. The history of attention deficit hyperactivity disorder. **ADHD Atten Def Hyp Disord** , v. 2, p. 241-255, 2010;

2. Haavik, J. et al. Clinical assessment and diagnosis of adults with attention-deficit/hyperactivity Disorder. **Expert Rev. Neurother**., v. 10, n. 10, p. 1569-1580, 2010;

3. Lundervold, A. J. et al. Attention Network Test in adults with ADHD - the impact of affective fluctuations. **Behavioral and Brain Functions**, v. 7, n. 27, p. 1-8, 2011;

4. Ranby, K. W. et al. Understanding the Phenotypic Structure of Adult Retrospective ADHD Symptoms During Childhood in the United States. **Clin Child Adolesc Psychol.**, v. 41, n. 3, p. 261-274, 2012;

5. Mattos, P.; Coutinho, G. Quality of life and ADHD. **J. Bras. Psiquiatr.**, v. 56, n. 1, p. 50-52, 2007;

6. Lopes, R. M. F. et al. Assessment of the attention deficit hyperactivity disorder (ADHD) in adults: a literature revision. **Avaliação Psicológica**, v. 4, n. 1, p. 65-74, 2005;

*7.* ARRUDA, M. et al. Are psychostimulants overprescribed in Brazilian school-aged children? A nationwide study. In: 3rd International Congress on ADHD, 2011, Berlin, Germany. Disponível em < <http://www.aprendercrianca.com.br/todas-as-categorias-dp461> >. Acesso em: 05 de setembro de 2012;

8. Arnsten, A. F. T.; Li, B. Neurobiology of Executive Functions: Catecholamine Influences on Prefrontal Cortical Functions. **Biol Psychiatry**, v. 57, p. 1377-1384, 2005;

9. MESQUITA, C. et al. Neuropsychological profile of adults with attention deficits: differences between ADHD and clinical controls. **Rev Psiq Clín.**, v. 37, n. 5, p. 212-215, 2010;

10. PUIG, M. V.; GULLEDGE, A. T. Serotonin and prefrontal cortex function: neurons, networks, and circuits. **Molecular Neurobiology**, 2011. No prelo;

11. SPENCER-SMITH, M.; ANDERSON, V. Healthy and abnormal development of the prefrontal cortex. **Developmental Neurorehabilitation**, v. 12, n. 5, p. 279-297, 2009;

12. BROWN, T. E. Executive Functions and Attention Deficit Hyperactivity Disorder: Implications of two conflicting views. **International Journal of Disability, Development and Education**, v. 53, n. 1, p. 35–46, 2006;

13. BOLFER. C. P. M. Avaliação Neuropsicológica das funções executivas e da atenção em crianças com transtorno do déficit de atenção/hiperatividade (TDAH). 2009. 113 f. Dissertação (Mestrado em Neurologia) – Faculdade de Medicina, Universidade de São Paulo, São Paulo. 2009;

14. STAGG, C. J.; NITSCHE, M. A. Physiological basis of transcranial direct current stimulation. **Neuroscientist**, v. 17, p. 37-53, 2011;

15. BRUNONI, A. R. et al. Clinical research with transcranial direct current stimulation (tDCS): challenges and future directions. **Brain Stimulation**, 2011. No prelo;

16. DASILVA, A. S. et al. Electrode Positioning and Montage in Transcranial Direct Current Stimulation. **J. Vis. Exp.**, v. 51, p. 1-11, 2011;

17. BRANDÃO, L. A.; DOMINGUES, R. C. Introduction and technique. In. BRANDÃO, L. A. (Org.). **MR Spectroscopy of the Brain**. Rio de Janeiro: Revinter, 2003. p. 2-17;

18. ________ . Psychiatric Disorders. In. BRANDÃO, L. A. (Org.). **MR Spectroscopy of the Brain**. Rio de Janeiro: Revinter, 2003. p. 182-189;

19. AGARWAL, R. et al. The Quality of Life of Adults with Attention Deficit Hyperactivity Disorder: A Systematic Review. **Innov Clin Neurosci.**, v. 9, n. 5–6, p. 10-21, 2012;

20. KOOIJ, S. J. et al. European consensus statement on diagnosis and treatment of adult ADHD: The European Network Adult ADHD. **BMC Psychiatry**, v. 10, n. 67, p 1- 24, 2010;

21. GRAHAM, J. et al. European guidelines on managing adverse effects of medication for ADHD. **Eur Child Adolesc Psychiatry**, v. 20, p. 17-37, 2011;

22. HABEL, L. A. et al. ADHD Medications and Risk of Serious Cardiovascular Events In Young and Middle-Aged Adults. **JAMA**, v. 306, n. 24, p. 2673-2683, 2011;

23. MATTINGLY , G. et al. Attention Deficit Hyperactivity Disorder Subtypes and Symptom Response in Adults Treated With Lisdexamfetamine Dimesylate. **Innov Clin Neurosci.**, v. 9, n. 5-6, p. 22-30, 2012;

24. SEPÚLVEDA, D. R. et al. Misuse of Prescribed Stimulant Medication for ADHD and Associated Patterns of Substance Use: Preliminary Analysis Among College Students. **J Pharm Pract.**, v. 24, n. 6, p. 551-560, 2011;

25. BRASIL, Ministério da Saúde. Resolução 196/96 do Conselho Nacional de Saúde/MS sobre diretrizes e normas regulamentadoras de pesquisa envolvendo seres humanos. **Diário Oficial da União**, 10 de outubro de 1996.
