# Supplementary material for: A Randomized, Double-Blind, Sham-Controlled Trial of Transcranial Direct Current Stimulation in Attention-Deficit/Hyperactivity Disorder
Source: PLoS One. 2015 Aug 12;10(8):e0135371. doi: 10.1371/journal.pone.0135371 (PMC4534404; doi:10.1371/journal.pone.0135371)
Supplement: S2 Protocol — (DOCX) [file pone.0135371.s004.docx]

| **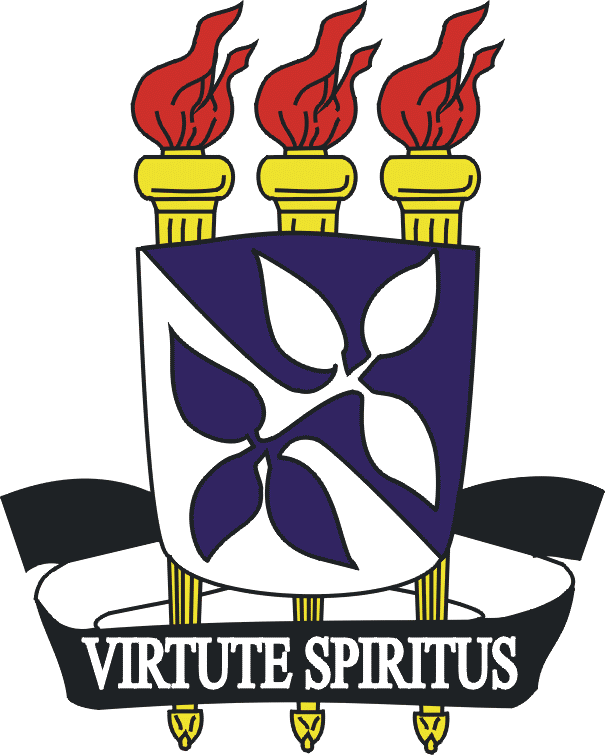** | **FEDERAL UNIVERSITY OF BAHIA**  **INSTITUTE OF HEALTH SCIENCES**  **POSTGRADUATE PROGRAM - INTERACTIVE PROCESS OF**  **ORGANS AND SYSTEMS** | 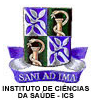 |
| --- | --- | --- |

**NEUROBIOLOGICAL ASPECTS OF ATTENTION DEFICIT–HYPERACTIVITY DISORDER: CONTRIBUTION OF TRANSCRANIAL DIRECT CURRENT STIMULATION ON INHIBITORY CONTROL**

**CAMILA SOUZA ALVES COSMO**

Project submitted to the Research Ethics Committee of the

Maternidade Climério de Oliveira- Institutional Review Board,

Federal University of Bahia, for consideration and appreciation,

developed under the guidance of Prof. Dr. Eduardo Pondé de Sena.

**Salvador, 2012**

**TABLE OF CONTENTS**

| INDEX OF ABBREVIATIONS AND ACRONYMS | 03 |
| --- | --- |
| I. OBJECTIVES | 04 |
| I. a. GENERAL | 04 |
| I. b. SPECIFIC | 04 |
| II. THEORETICAL | 04 |
| III. MATERIAL AND METHODS | 07 |
| IV. ETHICAL ASPECTS | 11 |
| V. TECHNICAL, SCIENTIFIC AND FINANCIAL FEASIBILITY | 11 |
| VI. RELEVANCE | 12 |
| VII. SCHEDULE | 12 |
| VIII. REFERENCES | 12 |

**INDEX OF ABBREVIATIONS AND ACRONYMS**

AAQoL- Quality of Life Questionnaire in Adults with ADHD;

ABENEPI- Brazilian Association of Neurology, Psychiatry and Allied Professions;

ADHD- Attention Deficit Disorder and Hyperactivity;

APB- Psychiatric Association of Bahia;

ASRS- Adult Self-Report Scale;

CPC-SSP- Research Center Clinical Sanatorium of São Paulo;

DSM-IV-TR- Diagnostic and Statistical Manual of Mental Disorders, Fourth Edition, Text Revision;

EEG- Electroencephalography;

ICF- Informed consent form;

mA- Miliampère;

MINI-plus- Mini International Neuropsychiatric Interview Plus;

MMSE- Mini-Mental State Examination;

QEEG- Quantitative electroencephalography;

SNB- Neurology Society of Bahia;

tDCS- Transcranial Direct Current Stimulation.

**I. OBJECTIVES**

I.a. General

To assess the neurobiological aspects associated with attention deficit-hyperactivity disorder (ADHD) and the contribution of transcranial direct current stimulation (tDCS) on inhibitory control.

I.b. Specific

I.b.1. To assess the contribution of transcranial direct current stimulation, comparing it to sham intervention, on the modulation of inhibitory control in adults with ADHD through Go/ No go task;

I.b.2. To examine the neurobiological aspects evidenced through the brain electrical activity observed by power analysis of frequency bands in patients with ADHD, using quantitative electroencephalography;

I.b.3. Proceed the Reconstruction of Functional Brain Network, of each participant, based on the quantitative EEG data through mathematical-computational modeling;

I.b.4. To register indicators of quality of life in adults with attention deficit-hyperactivity disorder through the Quality of Life Questionnaire for Adults with ADHD (AAQoL);

**II. THEORETICAL**

Human development includes differents stages of social and environmental exposure influenced by physiological and cultural aspects building the psychic and physical scope of an individual. In this context, attention deficit-hyperactivity disorder (ADHD) as a condition expressed by hyperactivity, impulsivity and reduced sustained attention, may contribute to the impairment of the social and emotional development of the individual, if there is no an early and effective intervention [1,2].

Attention deficit-hyperactivity disorder presents prevalence rates, in American studies, of 5 to 9% in childhood, especially in school age and between those, 67% still present the symptoms in adulthood, what may compromises the psychosocial, professional and emotional development [3- 6]. In a recent national study, Arruda et al observed a prevalence of ADHD among children and adolescents of 4.4% [7]. However, despite of the significant social impact, some of these patients remain undiagnosed and therefore without proper therapeutic intervention. The diagnosis is based on clinical assessment and identification of symptom established by the Diagnostic and Statistical Manual of Mental Disorders, Fourth Edition, Text Revision (DSM-IV-TR), as inattention, hyperactivity, impulsivity and general criteria [1 - 4, 6].

The clinical presentation possibly arises from disorders in the prefrontal cortex and its subcortical connections [1, 6, 8]. This assumption is supported by the hypothesis that it is a neurobiological disorder, evidenced by functional deficit in neurotransmitters such as dopamine and norepinephrine, and by disorders in the frontal lobe, triggering the symptoms observed in ADHD patients as impulsivity, difficulty to inhibit distractors behaviors, to plan and execute tasks under focused attention and to keep concentration [6]. These observations evidence the impairment of important executive functions in individuals with ADHD [2-4, 6, 8, 9].

Prefrontal cortex is the main brain area responsible for executive functions [10]. These functions correspond to neurocognitive skills of planning and execution of intentional and self-organized actions [8, 9, 11]. Considering these executive domains, inhibitory control is one of the most affected aspects in attention deficit-hyperactivity disorder [6]. It corresponds to the ability to inhibit inappropriate or inconvenient behavior and/ or inhibiting response to distractors stimuli [6, 8, 12]. A disorder of inhibitory control, thus significantly affects the social, emotional and cognitive performances of the individual, either by prevent the discontinuation of an inappropriate action or for not intervening in the interruption of a stimulus that discontinue appropriate responses [2, 3, 6].

There is evidence of a physiological association between the prefrontal cortex and inhibitory control [8, 12, 13]. Changes on this anatomical structure may account for an impairment in inhibitory control, evidenced behaviorally by actions that reflect inconsequence, unpredictability, intolerance to waiting, quick and inconsistent responses, possible exposure to hazards, and acceptance of multiple responsibilities and tasks simultaneously with consequent further withdrawals [8, 13].

This anatomical relationship and understanding of the neurobiological mechanisms comprises a physiological framework that supports the hypothesis that activation of the prefrontal area will result in a larger and more suitable inhibitory control, minimizing the socio-affective consequences associated to this condition.

Transcranial direct current stimulation is a simple and well known neuromodulatory technique [14, 15], that uses low voltage electrical currents to increase or decrease neuronal excitability of the stimulated area [14, 16]. This is a noninvasive and safe stimulation, having infrequent and mild adverse effects characterized by local discomfort, itching, paresthesia and/ or headache, with short lasting [16]. The application of this technique and the assessment of its effects will be performed by clinical observation considering as parameters a reducing on symptomatology evaluated through neuropsychological task- Go/ No go, and by neurophysiological assessments as quantitative electroencephalography (QEEG) and reconstruction of brain networks. In this trial, these techniques will assess possible prefrontal cortex activation after tDCS, and will evaluate the hypothesis of increase on inhibitory control and its behavioral consequences. It will be applied anodal stimulation at 1mA, aiming to enhance the excitability of the dorsolateral prefrontal cortex, according to the anatomical and physiological correlation mentioned above. At the end, it is expected a better adjustment of the behavioral responses associated to inhibitory control that compromise, ultimately, the quality of life of ADHD individuals [16].

The concept of quality of life is complex, wide and can be understood in different contexts: biological, economical, cultural and social [17]. Considering the approach of this study- the biological perspective, means quality of life as the condition of the individuals understand the disease, its challenges and behavioral inadequacies in a clear way that enables them to define adaptation strategies.

Regarding treatment approaches, there are pharmacological resources such as methylphenidate and lisdexamfetamine, that contribute to a better symptomatical control [18]. However, these drugs present important side effects, with unpredictable occurrence, such as insomnia, appetite loss, headache, muscle spasms, irritability, anxiety, cardiovascular events, among others, which may eventually result in discontinuation of the treatment [18- 22]. In this context, new therapeutic strategies with a simple and safe profile as transcranial direct current stimulation are essential.

The aim of this trial is to investigate the efficacy of tDCS over the prefrontal cortex on the modulation of inhibitory control in adults with ADHD, evidenced by behavioral and neurophysiological parameters previously mentioned. We expect to observe an increase of inhibitory response in ADHD individuals, that may result in a greater ability to inhibit inappropriate behaviors through the application of a low cost, safe and non-invasive technique.

**III. MATERIALS AND METHODS**

**III.a. Study Design**

This is a parallel and cross-sectional study which will include descriptive and analytical steps designed to investigate the neurobiological aspects of patients with attention deficit-hyperactivity disorder, and the response of these parameters, associated to inhibitory control, to the modulation by transcranial direct current stimulation.

**III.b. Population**

Patients with attention deficit hyperactivity disorder will be recruited by letter and email to the Psychiatric Association of Bahia (APB), Bahia Neurology Society (SNB) and the Brazilian Association of Neurology, Psychiatry and Allied Professions (ABENEPI) - chapter Bahia. We will also contact by mail and telephone, professional experts in ADHD, inviting them to refer potential patients for the study. We will also publish advertisements on the internet and on social networks, from October 2012 to December 2012, according to the inclusion criteria.

III.b.1. Inclusion criteria

-Ability to understand and sign the informed consent form;

-Diagnosis of attention deficit-hyperactivity disorder according to DSM-IV-TR and Adult Self-Report Scale (ASRS);

-Residents in Bahia;

-18 years old or older;

III.b.2. Exclusion criteria

-Major psychiatric disorders such as schizophrenia and bipolar disorder;

-Inability to understand the questionnaires applied (cognitive impairment- score ≤ 24 on the Mini-Mental State Examination) or illiterate;

-Abuse of psychoactive substances or alcohol, except nicotine and caffeine, during the last 12 months.

III.b.3. Sample size

This will be a randomized sample of ADHD individuals recruited through Internet media, social networks, and contact with ADHD experts and specialized societies of neurology, psychiatry and related fields, during the study period. A sample size of 50 subjects was calculated assuming a difference in proportion between the active tDCS and sham control groups of 40% on the Go/ No go performance, before and after interventions.

This calculation was performed using the statistical program STATA 12.0, considering an alpha of 0.05 and a power of 0.80, resulting in an sample of 25 subjects in each group, intervention and control. To address unexpected factors, we applied a drop-out rate of 20%, reaching a total sample size of 60 individuals.

**III.c. Study Variables**

III.c.1. Dependent variables

The following variables will be evaluated: neuropsychological outcome (Go/ No tasks); power analysis of frequency bands through QEEG; and brain reconstruction network model of each individual by QEEG.

III.c.2. Independent variables

Transcranial direct current stimulation and sham procedure.

**III.d. Procedures**

After screening through psychiatric interview, explanation about all steps of this study, and agreement expressed through signed informed consent form, ADHD patients will underwent a cognitive evaluation through the Mini-mental state examination (MMSE), Mini International Neuropsychiatric Interview Brazilian (MINI PLUS), Adult Self-Report Scale (ASRS), Quality Questionnaire life in Adults with ADHD (AAQoL), and an interview through a questionnaire. This evaluation script is an instrument based on the ADHD diagnostic criteria from DSM-IV-TR, and consists on epidemiological and clinical questions.

Individuals will be divided into two groups by block randomization, aiming at a balanced distribution between the groups (30 subjects each). Blocks shall be composed of 10 subjects, totaling six blocks. To compose each group will be called 5 individuals from each block.

Following it, the brain electrical activity will be recorded by EEG with 32 channels placed on the scalp acoording to the 10-20 EEG international system, during 5 minutes. The recording will be in a resting state, the first minute with eyes open looking for a fixed point, and the last 4 minutes with eyes closed. Then, participants will be asked to perform tasks on the computer: two Go / No go tasks adapted from the original version, the first using as a target a fruit and the second a letter. After the cognitive tasks, subjects will undergo active or sham stimulation, according to the previous randomization.

Participants of the intervention group will be submitted to tDCS at 1mA, anodal electrode over the left dorsolateral prefrontal cortex and cathodal electrode over the equivalent area on the right side. On control group subjects (sham group), electrodes will be placed at identical positions and sham stimulation will be applied. The stimulation device will turn on for only 30 seconds so that the patient will feel the initial sensation, then shutting down. The intervention and sham procedure will take place in one single session of 20 minutes to evaluate the immediate effect.

At the end of the intervention, subjects will perform again two Go/ No go tasks (randomly selected to avoid learning effect), and they will undergo another EEG recording, with the same previous technical caracteristics.

This study will be performed at Laboratory of Functional Electrostimulation at Federal University of Bahia.

**III.e. Statistical Analysis**

**Step 1**: socio-demographic, clinical and epidemiological description of the groups, using the usual procedures of descriptive statistics such as calculation of frequencies, measures of central tendency and dispersion;

**Step 2**: characteristics will be compared between groups at the baseline, using one-way analysis of variance (ANOVA) for continuous variables and chi-square test for categorical variables;

**Step 3**: Shapiro-Wilk test will be performed to assess normality assumption of the outcome variables;

**Step 4**: analysis of paired and independent samples using the t-test for comparison within each group and between intervention groups, or applying equivalent non-parametrics tests (according to Shapiro-Wilk test results), before and after intervention;

**Step 5**: Person's chi-square to evaluate blinding effectiveness, comparing between groups.

These analysis will be performed through Stata, version 12.0 for Windows. Results will be considered statistically significant if p ≤ 0.05.

**III.f. Study limitations and strategies to avoid bias**

III.f.1. **Selection bias**: it will be used objective criteria and already widely applied for the diagnosis of attention deficit-hyperactivity disorder, then it is unlikely the possibility of inclusion of individuals with other diagnosis.

III.f.2. **Measurement bias**: the selected scales are instruments with recognized and acceptable validity and reliability.

**IV. ETHICAL ASPECTS**

The present study strictly follows the ethical principles in research involving human subjects, according to the Declaration of Helsinki. All participants will be informed about the nature of the study and the all procedures. It will be included only those that sign informed consent form, in accordance with Resolution 196/96 of the National Health Council (Brazil) [23]. The signature of a witness will be required considering those patients unable to sign the free and informed consent form.

**V. TECHNICAL, SCIENTIFIC AND FINANCIAL FEASIBILITY**

The purpose of this study is to assess neurobiological aspects associated with attention deficit-hyperactivity disorder and the contribution of transcranial direct current stimulation to modulate inhibitory control and neurophysiological parameters, demonstrated by neurocognitive tasks (Go/ No go), QEEG and brain reconstruction network model. This study will not interfere with usual treatment of ADHD individuals, to avoid bringing any harm to those due treatment interruption. Through the investigation of the effectiveness of a simple technique, applied over the prefrontal cortex, it is expected to increase the inhibitory control in ADHD individuals. We also expect that the present trial will contribute scientifically to the development of neurophysiological assessment methods, for the study of ADHD, and to the evaluation of the applicability of a low cost, non-invasive and safe technique for otimization of inhibitory response in this population.

The project's budget has been reviewed and approved by Prof. Dr. Eduardo Pondé, and include costs of materials used in the data collection. The laboratory where the data collection will be held, already has the equipment needed as tDCS devices, 32 channels EEG, computers, besides electrodes, cables and sponges, being estimated only a very low expense with materials such as questionnaires, which explains the low budget and reiterates its financial feasibility.

**VI. RELEVANCE**

The results of this clinical trial will allow us to evaluate behavioral and neurobiological aspects of the attention deficit-hyperactivity disorder, besides to observe socio-demographical and clinical-epidemiological parameters in this population.

It will also be assessed, as mentioned previously, a low cost, simple and non-invasive technique for the modulation of the inhibitory control.

To the best of our knowledge, this trial will be the first study to assess the cognitive and neurophysiological effects of tDCS on ADHD patients. In the long term, we expect that our results might reinforce comprehensive programs of intervention and multidisciplinary approach in patients with ADHD.

**VII. SCHEDULE**

After the project approval, by this Institutional Review Board, the recruitment will start on May 2013, with data collection from June 2013. It is expected that the initial data analysis occurs approximately on March 2014, followed by elaboration of the thesis and scientific papers, during the second semester of 2014.

**VIII.** **REFERENCES**

1. Lange, K. W. et al. The history of attention deficit hyperactivity disorder. **ADHD Atten Def Hyp Disord** , v. 2, p. 241-255, 2010;

2. Haavik, J. et al. Clinical assessment and diagnosis of adults with attention-deficit/hyperactivity Disorder. **Expert Rev. Neurother**., v. 10, n. 10, p. 1569-1580, 2010;

3. Lundervold, A. J. et al. Attention Network Test in adults with ADHD - the impact of affective fluctuations. **Behavioral and Brain Functions**, v. 7, n. 27, p. 1-8, 2011;

4. Ranby, K. W. et al. Understanding the Phenotypic Structure of Adult Retrospective ADHD Symptoms During Childhood in the United States. **Clin Child Adolesc Psychol.**, v. 41, n. 3, p. 261-274, 2012;

5. Mattos, P.; Coutinho, G. Quality of life and ADHD. **J. Bras. Psiquiatr.**, v. 56, n. 1, p. 50-52, 2007;

6. Lopes, R. M. F. et al. Assessment of the attention deficit hyperactivity disorder (ADHD) in adults: a literature revision. **Avaliação Psicológica**, v. 4, n. 1, p. 65-74, 2005;

*7.* ARRUDA, M. et al. Are psychostimulants overprescribed in Brazilian school-aged children? A nationwide study. In: 3rd International Congress on ADHD, 2011, Berlin, Germany. Disponível em < <http://www.aprendercrianca.com.br/todas-as-categorias-dp461> >. Acesso em: 05 de setembro de 2012;

8. Arnsten, A. F. T.; Li, B. Neurobiology of Executive Functions: Catecholamine Influences on Prefrontal Cortical Functions. **Biol Psychiatry**, v. 57, p. 1377-1384, 2005;

9. MESQUITA, C. et al. Neuropsychological profile of adults with attention deficits: differences between ADHD and clinical controls. **Rev Psiq Clín.**, v. 37, n. 5, p. 212-215, 2010;

10. PUIG, M. V.; GULLEDGE, A. T. Serotonin and prefrontal cortex function: neurons, networks, and circuits. **Molecular Neurobiology**, 2011. No prelo;

11. SPENCER-SMITH, M.; ANDERSON, V. Healthy and abnormal development of the prefrontal cortex. **Developmental Neurorehabilitation**, v. 12, n. 5, p. 279-297, 2009;

12. BROWN, T. E. Executive Functions and Attention Deficit Hyperactivity Disorder: Implications of two conflicting views. **International Journal of Disability, Development and Education**, v. 53, n. 1, p. 35–46, 2006;

13. BOLFER. C. P. M. Avaliação Neuropsicológica das funções executivas e da atenção em crianças com transtorno do déficit de atenção/hiperatividade (TDAH). 2009. 113 f. Dissertação (Mestrado em Neurologia) – Faculdade de Medicina, Universidade de São Paulo, São Paulo. 2009;

14. STAGG, C. J.; NITSCHE, M. A. Physiological basis of transcranial direct current stimulation. **Neuroscientist**, v. 17, p. 37-53, 2011;

15. BRUNONI, A. R. et al. Clinical research with transcranial direct current stimulation (tDCS): challenges and future directions. **Brain Stimulation**, 2011. No prelo;

16. DASILVA, A. S. et al. Electrode Positioning and Montage in Transcranial Direct Current Stimulation. **J. Vis. Exp.**, v. 51, p. 1-11, 2011;

17. AGARWAL, R. et al. The Quality of Life of Adults with Attention Deficit Hyperactivity Disorder: A Systematic Review. **Innov Clin Neurosci.**, v. 9, n. 5–6, p. 10-21, 2012;

18. KOOIJ, S. J. et al. European consensus statement on diagnosis and treatment of adult ADHD: The European Network Adult ADHD. **BMC Psychiatry**, v. 10, n. 67, p 1- 24, 2010;

19. GRAHAM, J. et al. European guidelines on managing adverse effects of medication for ADHD. **Eur Child Adolesc Psychiatry**, v. 20, p. 17-37, 2011;

20. HABEL, L. A. et al. ADHD Medications and Risk of Serious Cardiovascular Events In Young and Middle-Aged Adults. **JAMA**, v. 306, n. 24, p. 2673-2683, 2011;

21. MATTINGLY , G. et al. Attention Deficit Hyperactivity Disorder Subtypes and Symptom Response in Adults Treated With Lisdexamfetamine Dimesylate. **Innov Clin Neurosci.**, v. 9, n. 5-6, p. 22-30, 2012;

22. SEPÚLVEDA, D. R. et al. Misuse of Prescribed Stimulant Medication for ADHD and Associated Patterns of Substance Use: Preliminary Analysis Among College Students. **J Pharm Pract.**, v. 24, n. 6, p. 551-560, 2011;

23. BRASIL, Ministério da Saúde. Resolução 196/96 do Conselho Nacional de Saúde/MS sobre diretrizes e normas regulamentadoras de pesquisa envolvendo seres humanos. **Diário Oficial da União**, 10 de outubro de 1996.
